# Curcumin Modulates Nitrosative Stress, Inflammation, and DNA Damage and Protects against Ochratoxin A-Induced Hepatotoxicity and Nephrotoxicity in Rats

**DOI:** 10.3390/antiox10081239

**Published:** 2021-08-02

**Authors:** Consiglia Longobardi, Sara Damiano, Emanuela Andretta, Francesco Prisco, Valeria Russo, Francesco Pagnini, Salvatore Florio, Roberto Ciarcia

**Affiliations:** 1 Department of Mental, Physical Health and Preventive Medicine, University of Campania “Luigi Vanvitelli”, Naples, Largo Madonna delle Grazie 1, 80138 Napoli, Italy; consiglia.longobardi@unicampania.it; 2 Department of Veterinary Medicine and Animal Productions, University of Naples “Federico II”, Naples, Via Delpino 1, 80137 Napoli, Italy; emanuela.andretta@unina.it (E.A.); francesco.prisco@unina.it (F.P.); valeria.russo@unina.it (V.R.); florio@unina.it (S.F.); 3 Unit of Radiology, Department of Medicine and Surgery, University of Parma, Via Gramsci 14, 43126 Parma, Italy; f.pagnini90@gmail.com

**Keywords:** ochratoxin A, hepatotoxicity, nephrotoxicity, nitrosative stress, inflammation, DNA damage

## Abstract

Ochratoxin A (OTA) is a fungal toxin of critical concern for food safety both for human health and several animal species, also representing a cancer threat to humans. Curcumin (CURC) is a natural polyphenol that has anti-apoptotic, anti-inflammatory, and antioxidant effects. The aim of this study was to investigate the cytoprotective effect of CURC against OTA-induced nephrotoxicity and hepatotoxicity through the study of the nitrosative stress, pro-inflammatory cytokines, and deoxyribonucleic acid (DNA) damage. Sprague Dawley rats were daily treated with CURC (100 mg/kg b.w.), OTA (0.5 mg/kg b.w), or CURC with OTA by oral gavage for 14 days. Our results demonstrated that OTA exposure was associated with significant increase of pro-inflammatory and DNA oxidative-damage biomarkers. Moreover, OTA induced the inducible nitric oxide synthase, (iNOS) resulting in increased nitric oxide (NO) levels both in kidney and liver. The co-treatment OTA + CURC counteracted the harmful effects of chronic OTA treatment by regulating inflammation, reducing NO levels and oxidative DNA damage in kidney and liver tissues. Histology revealed that OTA + CURC treatment determinates mainly an Iba1+ macrophagic infiltration with fewer CD3+ T-lymphocytes in the tissues. In conclusion, we evidenced that CURC exerted cytoprotective and antioxidant activities against OTA-induced toxicity in rats.

## 1. Introduction

Ochratoxin A (OTA) is a toxic secondary metabolite generated by several filamentous chlorophenolic fungal species belonging to *Aspergillus* and *Penicillium genera* [1,2]. Unfortunately, it is a food contaminant present in a large variety of agricultural products, including cereals, cereal-based products [3], grapes [4], herbs, coffee, cocoa, tea, fish, pork, milk and its products, poultry, and eggs [5,6]. Additionally, OTA is a danger both for people exposed to food diets containing OTA and animals. In these latter, OTA causes a greater morbidity and a diminution in production, negatively affecting the reproduction, nutrition, and growth of animals [7,8]. OTA is mostly hepatotoxic and nephrotoxic, but it also exhibited immunotoxic, carcinogenic, genotoxic, and possibly neurotoxic effects [9,10]. The International Agency for Research on Cancer (IARC) defined it as a possible carcinogen for humans (group 2B) [11]. The last European Food Safety Authority (EFSA) evaluation in 2006 and the international experts of the World Health Organization (WHO) stated that OTA may cause DNA damage by acting as a direct genotoxic carcinogen or through indirect mechanisms [12,13,14]. However, even though the exact molecular events involved in the DNA damage are not completely understood, both oxidative and nitrosative stress have been involved in these processes. In fact, it is hypothesized that OTA can induce overexpression of inducible nitric oxide synthase (iNOS), the enzyme responsible for producing nitric oxide (NO) [15]. NO plays a pivotal role as a redox-based signaling mediator, modulating enzyme activities, cytokine networks, and promoting reactive nitrogen species formation. In excess, NO in the kidney and liver may act as a toxic radical and causes an increase in nitrite and nitrate levels and in nitrosative stress, with protein and DNA damage as well as lipid peroxidation, necrosis, and apoptosis [16,17].

Curcumin (CURC), a natural polyphenolic compound extracted from the rhizomes of the turmeric plant (*Curcuma longa*) [18], has powerful antioxidant properties [19]. Since CURC can suppress genotoxic effects caused by radiation and various agents [20,21], one of the main objectives of the present work was to evaluate the beneficial effect of CURC on nitrosative stress and DNA damage in OTA-exposed rats. In our previous works, we demonstrated the efficacy of CURC on the kidney and liver of rats treated for 14 days with OTA as an antioxidant and powerful free-radical scavenger [22,23].

In addition, CURC is well known for its anti-inflammatory properties [19,20,21,22,23,24]. Indeed, CURC reduces the levels of nuclear factor kappa-light-chain-enhancer of activated B cells (NF-κB) and the levels of pro-inflammatory cytokines, such as interleukin-1β (IL-1β), tumor necrosis factor-alpha (TNF-α), and interleukin-6 (IL-6) [25,26,27]. Since OTA increases inflammation [28,29], in this work, we also assessed whether CURC could mitigate inflammatory processes in kidney and liver of OTA-exposed rats.

**Figure 1:**
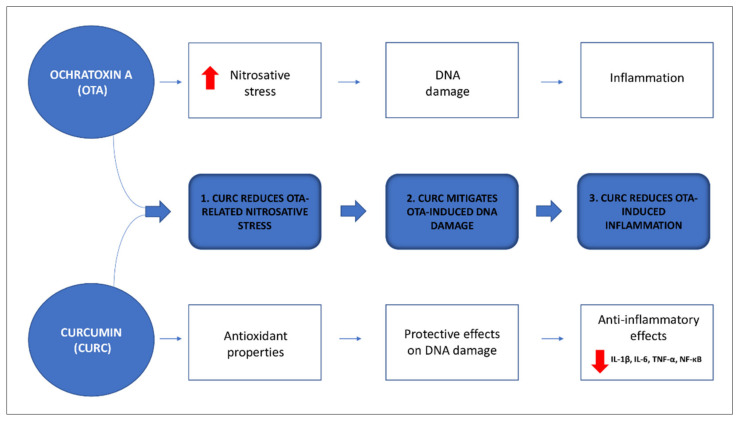
Schematic illustration of the working hypothesis of the present study.

A schematic illustration of working hypothesis of the present study is represented in Figure 1.

To understand the OTA-related negative consequences on animals and human health, it is firstly necessary to deepen the main mechanisms leading this mycotoxin to act. Since OTA primarily affects renal and hepatic tissues, we evaluated in this study the cytoprotective role of CURC against OTA-induced hepatic and renal toxicity.

The objective of the present work was to evidence for the first time the CURC cytoprotective action on OTA-induced toxicity in rats through the evaluation of the nitrosative stress, pro-inflammatory cytokines, and DNA damage, events that may be crucial in developing chronic OTA toxicity.

## 2. Materials and Methods

### 2.1. Chemicals

OTA and CURC were bought from Sigma-Aldrich (Milan, Italy) and 8-OHdG (8-hydroxy-2′-deoxyguanosine) ELISA kit from Stress Marq (Biosciences INC, Victoria, BC, Canada). Other chemicals and reagents used in this work were purchased from S.I.A.L s.r.l. (Rome, Italy). The animal supplier was Charles River Laboratories (Milan, Italy).

### 2.2. Ethic Statement

The experiment reported in this work shall abide by Italian and European law (National law: D.L. 26/2014; European law: Directive 2010/63/EU) and was approved by the Institutional Animal Care and Ethics Committee (Approval Number: 487/2018-PR).

### 2.3. Experimental Design

A total number of 24 Sprague Dawley rats (male, 250 to 270 g, 10 weeks old) were chosen for this study. All rats were randomly assigned (*n* = 6/group) to four groups and were maintained under environmentally controlled conditions (45–60% relative humidity, 22 ± 2 °C temperature; 12 h light–12 h dark cycle) and fed with a balanced commercial pellet (Teklad Global Diets) and tap water ad libitum. Rats were treated daily for 14 days by oral gavage as follows:

- Control group: the rats received 2 mL/kg b.w. of olive oil;
- OTA group: the rats received 0.5 mg/kg b.w. of OTA dissolved in 2 mL/kg b.w. of olive oil;
- CURC group: the rats received 100 mg/kg b.w. of CURC dissolved in 2 mL/kg b.w. of olive oil;
- OTA + CURC group: the rats received 2 mL/kg b.w. of olive oil containing 0.5 mg/kg b.w. of OTA plus 100 mg/kg b.w. of CURC.

Olive oil was used to achieve a greater stability of CURC. Both the duration of the experiment (14 days) and dosages of OTA and CURC were selected according to our previous data relating to their toxic chronic effects and antioxidant properties, respectively [30,31,32].

### 2.4. Samples Collection and Processing

After 2 weeks of daily treatment, animals were euthanized with 2% isoflurane (Isotec 4, Palermo, Italy), and after complete sedation, were sacrificed by cervical dislocation. Kidneys and liver of each rat were collected and washed with physiological saline 0.9%. To remove red blood cells and clots, the tissues were rinsed in 50 mmol/L of ice-cold sodium phosphate buffer saline (0.01 M, pH 7.4). Later, tissue samples were homogenized as follows: 1 g of the tissue for every 5–10 mL of ice-cold buffer solution, then centrifuged at 5000× *g* for 5 min. Then, the supernatants were kept at −80 °C for later evaluation of the content and activities of nitrosative stress and pro-inflammatory cytokines biomarkers in each tissue type. One-half of a kidney and a piece of liver were fixed in Bouin’s solution for histopathological and immunohistochemical analysis. Moreover, to measure oxidative DNA damage, 24-h urine was collected from the animals kept in metabolic cages. The results of lipid peroxidation and oxidative stress markers in kidneys and liver were reported in our previous papers [22,23], where CURC showed a good recovery of kidney and liver damage induced by OTA.

### 2.5. Pro-Inflammatory Cytokines Assay

Tumor necrosis factor-α (TNF-α), Nuclear factor-kappa B (NF-κB), interleukin-1β (IL-1β), and interleukin-6 (IL-6) levels in the hepatic and renal homogenates tissues were measured by commercial ELISA kits (Invitrogen, Waltham, MA, USA) following the manufacturer’s instruction. Optical densities (OD) obtained were read by ELISA Plate Reader (Glomax Multi detection system, Promega, Milan, Italy), the concentrations were expressed in nanograms per grams of tissue (ng/g tissue), and the measurements were conducted in triplicate.

### 2.6. Nitric Oxide Determination

NO was estimated spectrophotometrically in the hepatic and renal tissues with the Nitrite/Nitrate Assay Kit (Sigma-Aldrich, Milan, Italy) according to the manufacturer’s instructions. This method depends on the measurement of endogenous nitrite concentration as an indicator of NO production. It is based on the addition of Griess reagent, which converts nitrite into a deep purple azo compound, whose absorbance was read at 540 nm in a spectrophotometric multi-well plate reader (Glomax Multi detection system, Promega, Milan, Italy). The data were expressed as micromoles per gram of tissue (µmol/g tissue), and the measurements were conducted in triplicate.

### 2.7. iNOS Activity Assay

iNOS activities in liver and kidney tissues were determined by using a commercial ELISA kit (Sigma-Aldrich, Milan, Italy) in line with the manufacturer’s instructions. For this quantitative assay, we used the polyclonal iNOS rat specific antibody (Sigma-Aldrich, Milan, Italy) pre-coated onto microplates. Optical densities (OD) were read by ELISA Plate Reader (Glomax Multi detection system, Promega, Milan, Italy), and the results were expressed in nanogram per gram of tissue (ng/g tissue), and the measurements were conducted in triplicate.

### 2.8. 8-Hydroxy-2′-Deoxy Guanosine (8-OHdG) Analysis

Oxidative DNA damage was measured by the 8-OHdG ELISA kit from Stress Marq (Biosciences INC, Victoria, BC, Canada) in urine samples in accordance with the manufacturer’s protocol. Briefly, in a microtiter plate precoated with 8-OHdG, 50 μL of 8-OHdG standards (0.94–60 ng mL^−1^) and urine samples were separately plated in triplicates. Then, they were incubated with a specific HRP-conjugated antibody for 1 h. After the washing step, TMB substrate was incubated for 30 min. Then, the Stop solution provided by the kit reaction was used to stop the reaction. The absorbance at 450 nm was measured by spectrophotometer Glomax Multi Detection System (Promega, Madison, WI, USA). 8-OHdG urinary values were indicated as total amount excreted in 2 h, and the measurements were conducted in triplicate.

### 2.9. Hystopathological Examination

Twenty-four male Sprague Dawley rats (6 for group) were sacrificed, and their kidneys and livers were collected and fixed in Bouin’s solution for 24 h followed by trimming and routine paraffin wax embedding. Four-μm sections were used for hematoxylin and eosin staining, and three serial sections at 3 μm were stained for T-lymphocytes (CD3), B-lymphocytes (CD79), and macrophages (Iba1).

### 2.10. Immunoistochemistry Study

Immunohistochemistry was performed using our well-established method [33]. In detail, the liver and kidney paraffin sections at 3 μm were deparaffined in xylene and in decreasing series of alcohol. Peroxidases were blocked with a solution of hydrogen peroxide and methanol (4:1) for 15 min. The sections were incubated with a HIER citrate buffer pH 6.0 (Bio-Optica, Milan, Italy) for 20 min at 98 °C to perform antigen retrieval pretreatments. Subsequently, immunohistochemistry was carried out following the protocol suggested by the MACH1 Universal HRP-Polymer Detection Kit (Cat. No: M1U539 G, L10, Bio-Optica, Milan, Italy). The following primary antibodies were used: Polyclonal Rabbit Anti-CD3 (Code n. ab5690, Abcam, Cambridge, UK) at 1:100 in PBS, Monoclonal Mouse Anti-CD79 (Clone HM57, Dako, Santa Clara, CA, USA) at 1:100 in PBS, and Polyclonal Rabbit Anti-Iba1 (Code n. 019-19741, WAKO, Osaka, Japan) at 1:1000 in PBS [34]. Slides were examined and photographed with a light microscope (Nikon Eclipse E600 Tokyo, Japan) equipped with a microphotography system Nikon digital camera (DMX1200 Tokyo, Japan). A scoring system was designed to semi-quantitatively assess the degree of inflammation based on previously reported methods [35]. The number of inflammatory cells was scored by two independent pathologists (F.P. and V.R.) by light microscopy as: no infiltration (score 0); mild infiltration (score 1), on average 1 to 5 inflammatory cells per high-power field (HPF; 0.237 mm^2^; 40× objective and a 10× ocular with a field number of 22 mm); moderate infiltration (score 2), on average 6 to 10 inflammatory cells per HPF; and severe infiltration (score 3), on average more than 10 inflammatory cells per HPF. The average number was evaluated in at least 10 HPF for each sample.

### 2.11. Statistycal Analysis

The results on determination of pro-inflammatory cytokines activities were expressed as mean ± standard deviation (SD). Analysis of variance (ANOVA) tests followed by a Tukey’s test were used to analyze the differences (GraphPad Software 3.00, San Diego, CA, USA). Values of *p* < 0.05, *p* < 0.01, *p* < 0.001, and *p* < 0.0001 were considered statistically significant.

## 3. Results

### 3.1. Effect of CURC on Kidney and Hepatic Tissue Levels of NO

**Figure 2:**
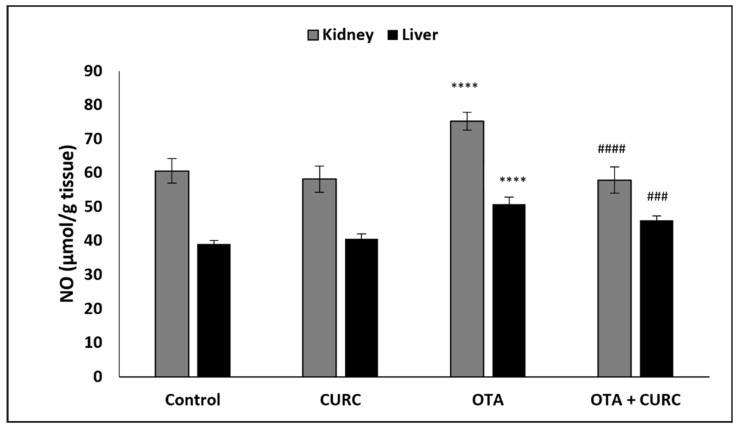
The antioxidant effect of curcumin (CURC) on the nitric oxide (NO) levels in the kidney and liver of different experimental groups after 14 days of treatment. Control group; CURC group (100 mg/kg b.w. of CURC); OTA group (0.5 mg/kg b.w. of OTA); OTA + CURC group (0.5 mg/kg b.w. of OTA plus 100 mg/kg b.w. of CURC). Data are expressed as mean ± standard deviation (SD) of *n*= 6 rats (**** *p* < 0.0001 vs. control; ^###^ *p* < 0.001 and ^####^ *p* < 0.0001 vs. OTA).

Figure 2 reports the results regarding renal and hepatic tissue levels of nitrite, stable products of the NO released in response to oxidative stress. The nitrite levels in kidney and liver were found significantly higher in OTA-treated group compared to the control group. However, co-administration with CURC significantly inhibited the increased production of nitrite in OTA group in both tissues. CURC-alone treatment did not exhibit a significant effect on nitrite release compared to the control group.

### 3.2. Effect of CURC on Liver and Kidney iNOS Activity

**Figure 3:**
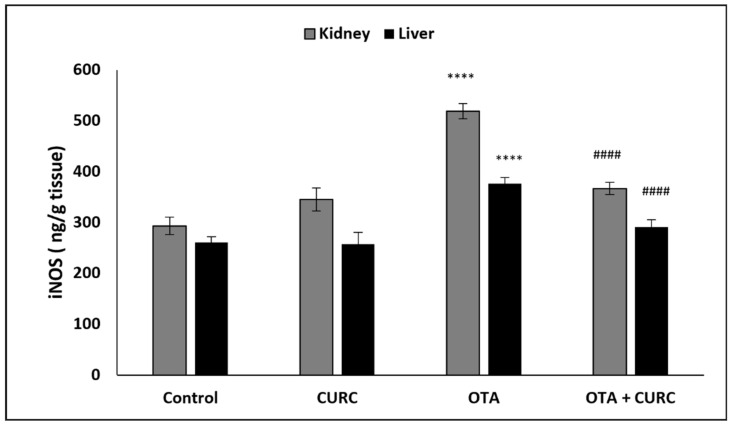
The antioxidant effect of curcumin (CURC) on kidney and liver inducible nitric oxide synthase (iNOS) activity in different experimental groups after 14 days of treatment. Control group; CURC group (100 mg/kg b.w. of CURC); OTA group (0.5 mg/kg b.w. of OTA); OTA + CURC group (0.5 mg/kg b.w. of OTA plus 100 mg/kg b.w. of CURC). Data are expressed as mean ± standard deviation (SD) of *n* = 6 rats in each group (**** *p* < 0.0001 vs. Control; ^####^ *p* < 0.0001 vs. OTA).

The hepatic and renal iNOS activity was significantly raised in OTA-treated group in comparison with the control group. However, co-administration with CURC significantly decreased the activities of hepatic and renal iNOS as compared to the OTA-treated group. CURC-alone treatment did not exhibit a significant effect on iNOS activity compared to the control group (Figure 3).

### 3.3. Effect of Curcumin on Pro-Inflammatory Cytokines Production in Kidney and Liver Tissues

**Figure 4:**
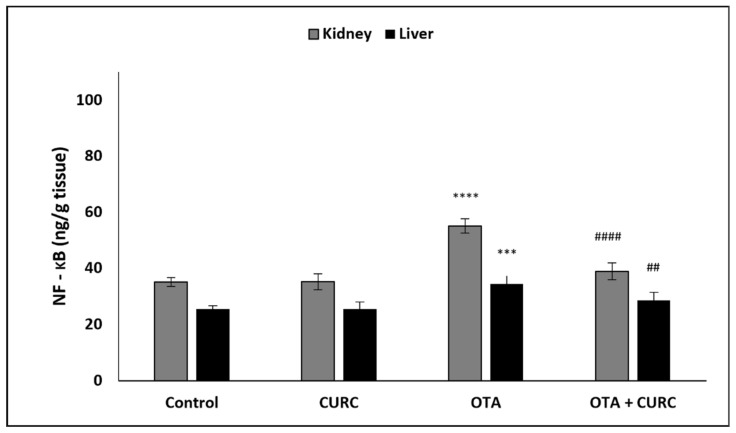
Effect of curcumin (CURC) on kidney and liver nuclear factor-kappa B (NF–kB) level in different experimental groups after 14 days of treatment. Control group; CURC group (100 mg/kg b.w. of CURC); OTA group (0.5 mg/kg b.w. of OTA); OTA + CURC group (0.5 mg/kg b.w. of OTA plus 100 mg/kg b.w. of CURC). Data are expressed as mean ± standard deviation (SD) of *n*= 6 rats in each group (*** *p* < 0.001 and **** *p* <0.0001 vs. control; ^##^ *p* < 0.01 and ^####^ *p* < 0.0001 vs. OTA).

**Figure 5:**
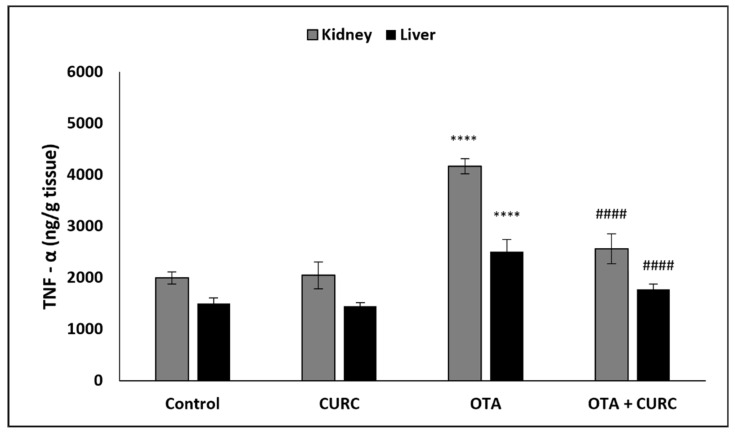
Effect of curcumin (CURC) on kidney and liver tumor necrosis factor-α (TNF-α) level in different experimental groups after 14 days of treatment. Control group; CURC group (100 mg/kg b.w. of CURC); OTA group (0.5 mg/kg b.w. of OTA); OTA + CURC group (0.5 mg/kg b.w. of OTA plus 100 mg/kg b.w. of CURC). Data are expressed as mean ± standard deviation (SD) of *n* = 6 rats in each group (**** *p* < 0.0001 vs. control; ^####^ *p* < 0.0001 vs. OTA).

**Figure 6:**
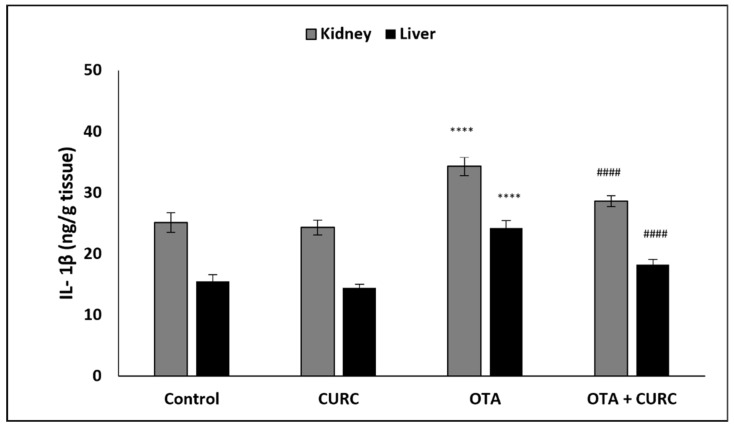
Effect of curcumin (CURC) on kidney and liver interleukin-1β (IL-1β) level in different experimental groups after 14 days of treatment. Control group; CURC group (100 mg/kg b.w. of CURC); OTA group (0.5 mg/kg b.w. of OTA); OTA + CURC group (0.5 mg/kg b.w. of OTA plus 100 mg/kg b.w. of CURC). Data are expressed as mean ± standard deviation (SD) of *n* = 6 rats in each group (**** *p* < 0.0001 vs. control; ^####^ *p* < 0.0001 vs. OTA).

**Figure 7:**
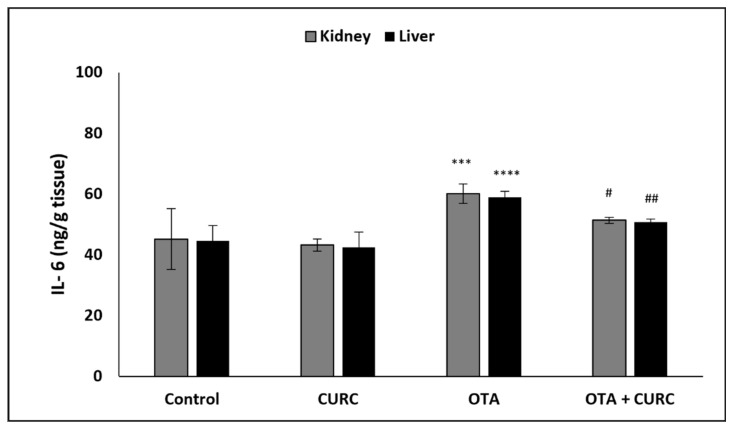
Effect of curcumin (CURC) on kidney and liver interleukin-6 (IL-6) level in different experimental groups after 14 days of treatment. Control group; CURC group (100 mg/kg b.w. of CURC); OTA group (0.5 mg/kg b.w. of OTA); OTA + CURC group (0.5 mg/kg b.w. of OTA plus 100 mg/kg b.w. of CURC). Data are expressed as mean ± standard deviation (SD) of *n* = 6 rats in each group. (*** *p* < 0.001 and **** *p* < 0.0001 vs. control; ^#^ *p* < 0.05 and ^##^ *p* < 0.01 vs. OTA).

In OTA-treated group compared to the control group, a significant increase of hepatic and renal NF-κB, TNF-α, IL-1β, and IL-6 levels was found. However, co-administration with CURC significantly modulated the values of renal and hepatic NF-κB, TNF-α, IL-1β, and IL-6 as compared to the OTA-treated group. There was no significant difference in the renal and hepatic NF-κB, TNF-α, IL-1β, and IL-6 levels between the control group and the CURC one (Figure 4, Figure 5, Figure 6 and Figure 7).

### 3.4. Effect of Curcumin on OTA-Induced DNA Damage

**Figure 8:**
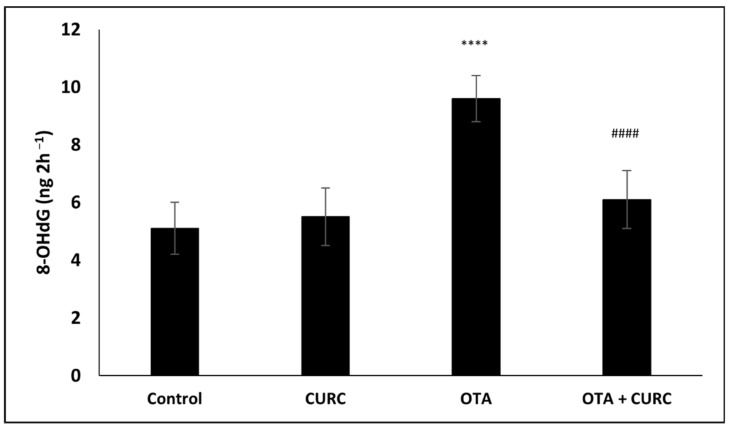
Effect of curcumin (CURC) on urinary 8-OHdG concentration at the end of treatment. Control group; CURC group (100 mg/kg b.w. of CURC); OTA group (0.5 mg/kg b.w. of OTA); OTA + CURC group (0.5 mg/kg b.w. of OTA plus 100 mg/kg b.w. of CURC). Data are shown as mean ± standard deviation (DS) (*n* = 6 for each group) and were compared by ANOVA (**** *p* <0.0001 vs. control, ^####^ *p* < 0.0001 vs. OTA).

The 8-OHdG levels were measured in urine samples of control, OTA, CURC, and OTA + CURC groups. Urinary 8-OHdG levels were significantly higher in the OTA group with respect to the control group. CURC treatment in OTA group at the end of treatment significantly reduced urinary 8-OHdG levels when compared to the OTA group (Figure 8).

### 3.5. Histopayhological Examination

**Figure 9:**
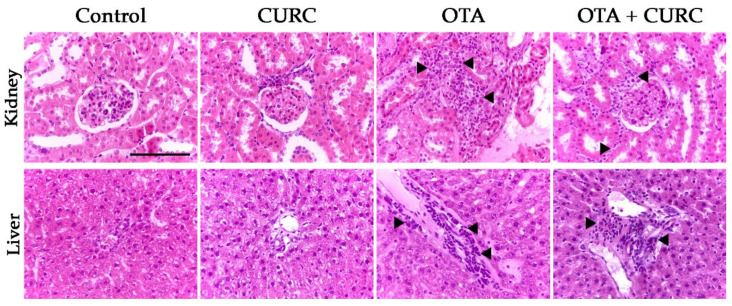
Histological examination of kidneys and liver of rats. Control group; CURC group (100 mg/kg b.w. of CURC); OTA group (0.5 mg/kg b.w. of OTA); OTA + CURC group (0.5 mg/kg b.w. of OTA plus 100 mg/kg b.w. of CURC). Hematoxylin and Eosin stain, 40× magnification, scale bar = 100 µm. Arrowheads indicate the infiltrating inflammatory cells.

**Figure 10:**
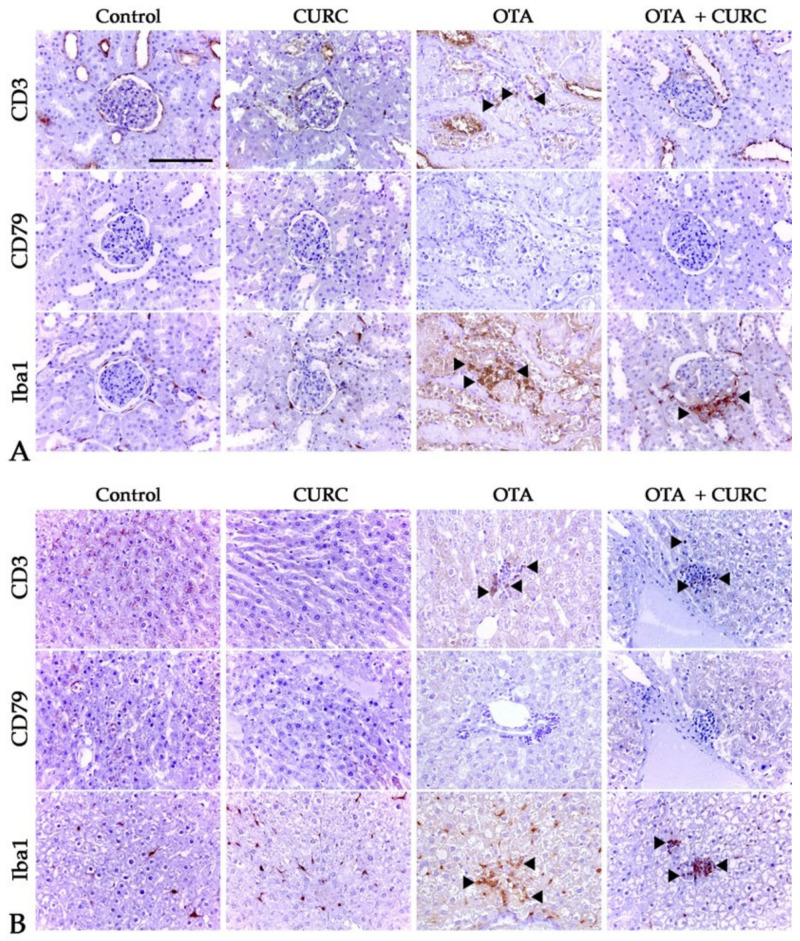
Immunohistochemical characterization of the inflammatory infiltrates in kidney (**A**) and liver (**B**) of rats. Control group; CURC group (100 mg/kg b.w. of CURC); OTA group (0.5 mg/kg b.w. of OTA); OTA + CURC group (0.5 mg/kg b.w. of OTA plus 100 mg/kg b.w. of CURC). Immunohistochemistry, 40× magnification, scale bar = 100 µm. Arrowheads indicate positive cells.

**Figure 11:**
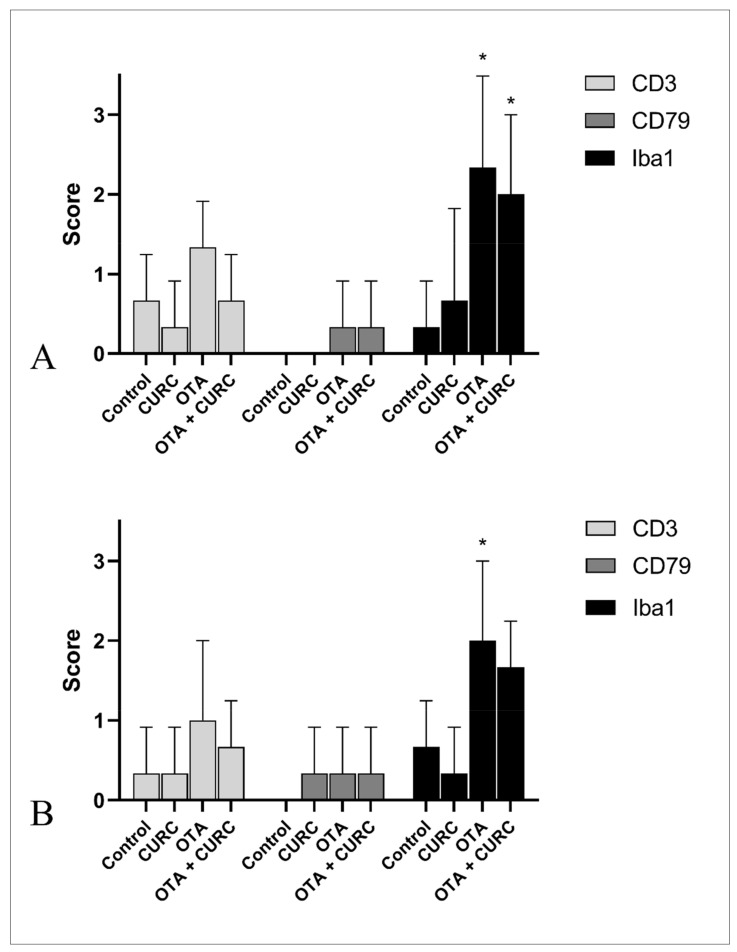
Semi-quantitative assessment of the inflammatory infiltrates in kidneys (**A**) and liver (**B**) of rats. Control group; CURC group (100 mg/kg b.w. of CURC); OTA group (0.5 mg/kg b.w. of OTA); OTA + CURC group (0.5 mg/kg b.w. of OTA plus 100 mg/kg b.w. of CURC). Iba1 antigen has been used to stain macrophages, CD3 antigen for T-lymphocyte, and CD79 antigen for B-lymphocyte (* *p* < 0.05 vs. control).

Inflammatory changes in the kidney of rats of both the OTA + CURC and OTA groups consisted of a multifocal interstitial inflammatory infiltrate, which occasionally invaded Bowman’s spaces and obscured the glomeruli. The inflammatory infiltrate was composed of mononuclear inflammatory cells. The liver of both the OTA + CURC and OTA group showed a multifocal interstitial inflammatory infiltrate often located in portal spaces and composed of mononuclear inflammatory cells. Only sparse infiltrating inflammatory cells were evident in the kidneys and liver of rats of the control and CURC groups (Figure 9). A semi-quantitative assessment of the morphological changes in both kidneys and liver were already reported in previously published papers [22,23] Here, we characterized the immunophenotype of the inflammatory cells. The inflammatory infiltrate is dominated by Iba1-positive macrophages associated with less CD3-positive T-lymphocytes and sparse CD79-positive B cells in both kidney and liver of rats belonging to the OTA + CURC and OTA groups (Figure 10 and Figure 11).

## 4. Discussion

OTA is a mycotoxin which causes serious health problems in both humans and animals, and we considered necessary in this work to investigate the main biochemical mechanisms of action underlying the toxic effects related to this mycotoxin. The primary target site for the action of OTA is the kidney [36] due to its slow elimination and vulnerability to oxidative damage caused by OTA-induced free radicals [37]. OTA also promotes inflammation, oxidative stress, or even cancer in the liver, which is the organ responsible for the processes of biotransformation of the mycotoxin [38]. The correlation between the impairment of oxidative balance and OTA-related toxicity is now in the public domain [9,10,39,40]. Therefore, the attention of the scientific community is directed towards the identification of natural antioxidant compounds with free-radical scavenging capacities to combat OTA-induced cytotoxicity. The purpose of this study was to assess if CURC, a natural polyphenol compound used as an herbal medicine for inflammatory diseases, had potential protective role on OTA-promoted nitrosative stress, DNA injury and, especially, inflammation in rat liver and kidney tissues. Our recent works [22,23] demonstrated the antioxidant effects of CURC on OTA-induced nephrotoxicity and hepatotoxicity in rats. In particular, while OTA caused oxidative stress, lipid peroxidation and reduced activity of the main antioxidant enzymes, CURC inhibited lipid peroxidation enhancing the activities of antioxidant enzymes in OTA-treated rats. Therefore, these data, in according with other findings present in the literature [41,42], demonstrated that CURC can be a good antioxidant and free-radical scavenger. The scavenging action of CURC against free radicals could be ascribed to the presence of methoxy and phenolic groups on the phenyl ring and 1,3-diketone [43], which make it capable of inhibiting lipid peroxidation. These mechanisms could explain at least in part the antioxidant effects of CURC. Starting from these data in this study, we investigated, for the first time, the cytoprotective and anti-inflammatory effect of CURC against OTA-induced toxicity in rats through the assessment of the nitrosative stress, pro-inflammatory cytokines, and oxidative DNA damage, processes that may result decisively in developing chronic toxicity connected to long-term exposure to OTA. Inflammatory processes and oxidative status impairment are both strongly linked to pathophysiological processes responsible for many chronic disorders, including liver and kidney diseases [44,45]. In fact, the altered oxidative balance can enhance chronic inflammation by the activation of different transcription factors, leading to inflammatory cytokines release. On the other hand, inflammation increases reactive nitrogen and oxygen species (RNS/ROS) levels, inducing further oxidation [46]. In this work, OTA treatment induced the overexpression of the inducible nitric oxide synthase (iNOS), causing a rise of NO levels in liver as well as in kidney and leading, in turn, to the nitrosative stress. NO is a pro-inflammatory mediator [47,48], and its overproduction can result in apoptosis and DNA or mitochondrial membrane damage [49]. The mechanism underlying NO oxidative toxicity may be dependent on the reaction between NO and superoxide radical to generate the pro-oxidant peroxynitrite, which is rapidly decomposed to the nitro radical, giving rise to nitrosative stress and DNA damage [50].

In particular, ROS and RNS promote DNA injury, developing 8-OHdG and 8-nitroguanosine and oxidative and nitrosative markers stress, respectively [51,52].

Since plasma is characterized by a huge complexity, urine represents a more used biological matrix to perform the evaluation of free 8-OHdG than plasma [53,54]. In the present work, we found an appreciable increase in 8-OHdG expression in urine of rats in the OTA-treated group. On the other hand, the co-treatment with CURC significantly reduced this 8-OHdG over-expression, exhibiting in this way the protective action of CURC against OTA-induced DNA damage.

Furthermore, OTA also increased the activity of NF-κB, in agreement with the data in the literature [15,17]. NF-κB regulates the immune response in many organs, playing a crucial role in various inflammatory diseases [55,56]. In fact, gene regulation and activation of proinflammatory cytokines, including IL-1β, TNF-α, IL-6, and iNOS, is controlled by NF-κB [57,58]. NF-κB dysregulation has been related to different pathological conditions, including cancer and several inflammatory diseases [59]. NF-κB signaling pathway could be activated or inactivated by different factors or stimulus signaling, such as TNF-α [60].

Moreover, TNF-α, a pro-inflammatory cytokine, represents a key mediator of inflammatory tissue damage [61,62]. Our results indicated the remarkable role of inflammatory processes in OTA-induced toxicity via NO, TNF-α, IL-6, and IL-1β elevations in renal and liver tissues of OTA-treated rats. The anti-inflammatory property of CURC observed in the rats simultaneously treated with OTA and CURC may be mediated through the ability of curcumin to suppress the pro-inflammatory cytokines TNF-α, IL-6, and IL-1β in renal and liver tissues. Ghosh et al. [63] suggested that the anti-inflammatory effects of curcumin are due to its ability to reduce TNF-α, IL-1, IL-6, COX-2, and NF-κB. CURC, with its ability to bind TNF-α directly, can inhibit both the production and activity of this cytokine [64]. As previously reported, histology confirmed the severity of inflammatory changes in both the kidney and liver of rats treated with OTA with respect to animals co-treated with both OTA and antioxidants such as CURC or anthocyanins [22,23,65]. In these tissues, the inflammatory infiltrate was characterized by the presence of mononuclear cells. Immunohistochemistry analysis evidenced that the infiltrate was dominated by Iba1+ macrophages with fewer CD3+ T-lymphocytes in OTA + CURC tissues. These findings are in agreement with the overexpression of the studied inflammatory mediators. Macrophages are a key population of innate immunity, and iNOS is a hallmark molecule of classical pathway-activated (M1) macrophages [66]. iNOS is highly expressed upon activation of the transcription factor NF-κB in response to many stimuli and regulates the differentiation and function of immune cells via nitration of key molecules involved in transcriptional or signaling pathways [66]. When activated, M1 macrophages express pro-inflammatory cytokines, such as IL-1β, IL-6, and TNF-α [67]. IL-1β and TNF-α serve a critical role in leukocytes recruitment by promoting adhesion of leukocytes of the endothelium and their migration through blood vessels [68]. These cytokines are mainly released by activated macrophages and dendritic cells; however, TNF-α is also produced by T-lymphocytes and mast cells, and IL-1 is produced by endothelial cells as well [69,70]. IL-6 is a two-faced cytokine with both anti-inflammatory and pro-inflammatory activity depending on the tissue microenvironment [35]. IL-6 is produced by macrophages, lymphocytes, and endothelial cells, and in an inflammatory microenvironment together with other proinflammatory cytokines, including TNF-α, IL-6 promotes inflammation [35].

In conclusion, the biochemical data, confirmed by histopathological examinations, showed the cytoprotective action of CURC on the renal and hepatic rat tissues through the mitigation of OTA-induced inflammation, nitrosative stress and oxidative DNA damage.

## Data Availability

The datasets used and/or analyzed during the current study are not publicly available but are available from the corresponding authors on reasonable request.
